# Pharmacokinetics of long-lasting, high-dose dexmedetomidine infusions in critically ill patients

**DOI:** 10.1186/cc9773

**Published:** 2011-03-11

**Authors:** T Iirola, R Aantaa, R Laitio, E Kentala, M Lahtinen, A Wighton, C Garratt, T Ahtola-Sätilä, KT Olkkola

**Affiliations:** 1 University of Turku and Turku University Hospital, Turku, Finland; 2 Orion Pharma, Nottingham, UK

## Introduction

The aim of this study was to characterize the pharmaco-kinetics of long dexmedetomidine (dexmed) infusions and assess the dose linearity of high doses.

## Methods

Dexmed was infused to critically ill intensive care patients for 12 hours using a constant infusion rate determined by the prestudy dose of propofol or midazolam. After the first 12 hours, the infusion rate of dexmed was titrated between 0.1 and 2.5 μg/kg/hour using prefixed levels to maintain sedation in range of 0 to -3 on the Richmond Agitation-Sedation Scale (RASS). Dexmed was continued as long as required to a maximum of 14 days. Safety and tolerability were assessed by adverse events, heart rate, blood pressure, ECG and laboratory tests.

## Results

**Figure 1:**
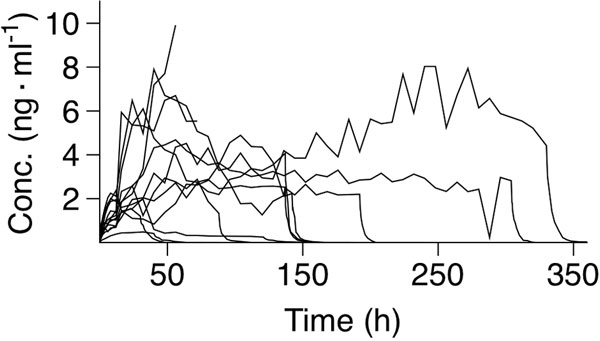
**Dexmedetomidine concentration profiles of the 13 patients**.

Dexmed concentration profiles of the 13 patients during the infusion and 48-hour follow-up are depicted in Figure 1. The geometric mean values (CV%) for length of infusion, dexmed half-time, clearance and volume of distribution (elimination) were 91 hours (117%), 3.7 hours (38%), 41 l/hour (47%) and 223 l (35%), respectively. There was a linear relationship (*r*^2 ^= 0.95; *P *< 0.001) between the areas under the dexmed plasma concentration-time curves and cumulative doses of dexmed. All but one patient needed propofol to keep the RASS value in the target zone. The most common adverse events were tachycardia, hypotension and hypertension.

## Conclusions

The pharmacokinetics of dexmed was linear up to the dose of 2.5 μg/kg/hour. Despite the high dose and long-lasting infusions, safety findings were as expected for dexmed and the patient population.

